# Assessment of Early Antibiotic Administration for Patients With Sepsis in the Emergency Department: A Retrospective Observational Study at a Tertiary Care Hospital

**DOI:** 10.7759/cureus.106896

**Published:** 2026-04-12

**Authors:** Abin Thomas, Sreehitha E P, Vishnu C Unnikrishnan, Ritvik Sajan, Anglin Vincent V, Sreekrishnan Trikkur, Gireesh Kumar, Sabarish Nair, Bharath Prasad S, Naveen Mohan

**Affiliations:** 1 Emergency Medicine, Amrita Institute of Medical Sciences and Research Center, Kochi, IND; 2 Pharmacy Practice, Amrita School of Pharmacy, Amrita Vishwa Vidyapeetham, Kochi, IND

**Keywords:** antibiotic administration, emergency room (er), morbidity and mortality, sepsis and septic shock, sepsis without septic shock, septic shock, time to antibiotic administration

## Abstract

Background

Sepsis is a severe medical illness brought on by the body’s exaggerated response to an infection. It develops when the body’s reaction to the infection results in extensive inflammation, which may lead to the potential risk of organ failure or damage. Sepsis is an acute health crisis that requires immediate treatment, inevitably in a hospital with antibiotics and supportive care. Early administration of broad-spectrum antibiotics is a cornerstone of sepsis management and is strongly recommended by the guidelines. Delays in antibiotic initiation are associated with worse clinical outcomes and increased mortality.

Objective

To evaluate the timing of antibiotic administration among patients presenting with septic shock and sepsis without septic shock in the emergency department and to assess its association with in-hospital mortality in relation to guideline recommendations.

Materials and methods

This retrospective observational study was conducted among 95 patients diagnosed with septic shock and sepsis without septic shock in the emergency department of a tertiary care hospital between February 2024 and July 2024. Data, including demographics, clinical parameters, laboratory findings, and time to antibiotic administration, were obtained from electronic medical records. Continuous variables were summarized as median (interquartile range or IQR), and categorical variables were analyzed using Pearson's chi-square test. A p-value <0.05 was considered statistically significant.

Result

Of the 95 patients included in the study, 65 (68.4%) had sepsis without septic shock and 30 (31.6%) had progressed to septic shock. Among the patients with septic shock treated for antibiotics, 67% received them within 60 minutes, 50% between 60-120 minutes, and 78.3% after 120 minutes. This association was statistically significant (χ²= 11.26, p=0.04). Subgroup analysis demonstrated a similar increasing trend in mortality with delayed treatment in patients with septic shock and sepsis without septic shock.

Conclusion

Delayed antibiotic administration was significantly associated with increased mortality in patients with septic shock and sepsis without septic shock. Although earlier treatment was more commonly observed in patients with septic shock, delays beyond the recommended timeframes were frequent. These findings highlight the importance of early recognition and prompt antibiotic initiation in emergency settings to improve patient outcomes.

## Introduction

The body’s overreaction to an infection or virus is termed sepsis and can lead to shock if not treated properly. Sepsis is a medical emergency that needs to be treated swiftly to avoid organ failure, tissue damage, and death. Individuals aged ≥65 years, infants less than one year, immunocompromised individuals, and those with chronic illnesses are at higher risk for sepsis [1]. According to the World Health Organization (WHO), in 2020, approximately 49 million sepsis cases and nearly 11 million sepsis-related deaths were reported [2]. It affects the low- and middle-income countries (LMICs), with high incidence and fatality rates due to delayed diagnosis and poor treatment protocols [3].

The pathophysiology of sepsis involves a complex interplay of infection and inflammatory response, which ultimately leads to organ damage and tissue dysfunction [4]. This initial infection in the body can trigger an excessive release of pro-inflammatory mediators, such as chemokines, cytokines, and other signaling molecules, which affect multiple organ systems. This inflammation, if not taken care of immediately, can progress to septic shock, characterized by widespread infection across body, low blood pressure, and organ failure [5].

The signs and symptoms commonly associated with sepsis includes one or more of the following: shivering, fever, or feeling very cold; extreme pain or discomfort throughout the body; clammy or discolored skin; confusion or disorientation; shortness of breath; and an elevated heart rate [6]. Investigations which can help in diagnosis of sepsis includes a complete blood count (CBC), serum lactic acid, arterial blood gas (ABG) analysis, and C-reactive protein (CRP) measurement. Blood, urine, and sputum cultures should also be performed in addition to blood sugar, liver function, renal function, prothrombin time (PT), international normalized ratio (INR), serum fibrinogen, pro-calcitonin level, and serum albumin. Additionally, imaging studies such as chest X-ray, ultrasound of abdomen (USG), electrocardiogram (ECG), and echocardiography (ECHO) are recommended [7]. These laboratory findings can be helpful for the early determination of sepsis and at a faster rate.

Early antibiotic administration is a crucial for the effective management of sepsis. The primary goal is to rapidly and effectively combat the underlying infection to prevent further complications and improve patient outcomes. Several studies have demonstrated that the risk of mortality is greatly increased when antibiotics are not administered within the first hour of sepsis being recognized. In patients diagnosed with septic shock, every hour of delay has been attributed to cause death in one-tenth of the population [8,9]. Early administration of broad-spectrum antibiotics is critical in sepsis management. Current Surviving Sepsis Campaign (SSC 2021) guidelines recommend initiation as soon as possible, ideally within one hour for septic shock and within three hours for sepsis, as delays are associated with increased mortality [10]. Studies conducted by Todi et al. and others signify that inefficient antibiotic introduction in sepsis is caused by difficulties in resource-constrained environments, involving congested emergency rooms, a lack of standardized triage methods, and inadequate staff training [11]. This emphasizes how critical it is to evaluate the timing of antibiotics and how they relate to patient outcomes in emergency settings.

Given this background, the purpose of our study was to evaluate the timing of antibiotic administration in patients with septic shock and sepsis without septic shock. Our objective was to assess the significant shortcomings in the management of emergency sepsis and contribute to the growing amount of data that supports early intervention for better prognosis.

## Materials and methods

Study design and setting

This was a retrospective observational study conducted to evaluate the impact of antibiotic timing in patients suffering from septic shock and sepsis without septic shock in the emergency department of a tertiary care hospital in a southern state of India. Patient data were obtained through the review of electronic medical records (EMR) and hospital databases.

The Institutional Ethics Committee (IEC) waived the requirement for ethical approval due to the retrospective nature of the study and the use of anonymized patient data. No identifiable or confidential patient information was collected during the study.

Study participants

Inclusion Criteria

Adult patients diagnosed with septic shock or sepsis without septic shock according to the sepsis-3 criteria [12], and patients who presented to the emergency department between February 2024 and July 2024.

Exclusion Criteria

Patients less than 18 years of age and pediatric patients. Patients with incomplete medical records, particularly missing information on the time of antibiotic administration, were also excluded.

The study was conducted over six months, from February 2024 to July 2024, in the Emergency and Critical Care Department of a tertiary care hospital in the southern part of India.

Objectives

The main objective of the study was to evaluate the relationship between the timing of antibiotic administration and clinical outcomes in patients diagnosed with septic shock and sepsis without septic shock. Specifically, the study aimed to determine whether earlier antibiotic administration had a statistically significant impact on patient mortality and to explore differences in outcomes between patients with septic shock and sepsis without septic shock.

Methodology

Patients who met the inclusion criteria were identified through the retrospective review of medical records of those presenting to the Emergency Room (ER) during the study period. Demographic and clinical details were extracted from the records, along with the time of antibiotic administration. Based on their clinical diagnosis, patients were categorized into two groups: 1) those with septic shock and 2) those with sepsis without septic shock. The study then analyzed the differences in timing and outcomes between these two groups. Also, blood cultures were obtained prior to the initiation of antibiotic therapy wherever feasible, in accordance with surviving sepsis campaign recommendations. However, antibiotic administration was not delayed in critically-ill patients if obtaining cultures caused significant delay. The statistical tests used in the study involved Mann-Whitney U test, used to compare continuous variables between the two groups (sepsis without septic shock and septic shock), and Pearson chi-square test to compare categorical variables between the groups.

Definitions

Sepsis

Sepsis is defined as a life-threatening organ dysfunction characterized by a dysregulated host response to infection. Patients in our study were diagnosed with sepsis based on a combination of clinical evaluation and objective assessment, performed using the Sequential Organ Failure Assessment (SOFA) score. The SOFA score is calculated by evaluating six organ systems in order to assess patient’s organ functioning. Respiratory function is measured using partial pressure of oxygen in arterial blood (PaO2)/ fraction of inspired oxygen (FiO2) or, alternatively via oxygen saturation to FiO2 ratio (SpO2/FiO2). Cardiovascular status determined by monitoring the patient's mean arterial pressure (MAP) and the need of vasopressor medications to maintain adequate blood pressure. Liver function is evaluated via the measurement of serum bilirubin levels, while renal function is assessed by measuring serum creatinine concentrations. Coagulation status is monitored by checking the patient’s platelet levels, and neurologic function is determined by the Glasgow Coma Scale (GCS) score [13].

Therefore, diagnosis of sepsis for all patients in our study was established using the SOFA score. An increase in SOFA score of two points or more from baseline, in patients with a suspected or confirmed infection, indicates worsening organ dysfunction, which is representative of sepsis [14].

Septic Shock

Septic shock is a subset of sepsis with an increased risk of mortality. Patients were classified as having septic shock, if they required vasopressor therapy to maintain a MAP of 65 mmHg or greater and had serum lactate levels rising above 2 mmol/L despite fluid resuscitation. Hyperventilation with respiratory alkalosis (low levels of partial pressure of carbon dioxide (PaCO2) and increase in arterial pH), along with an increase in serum lactate levels, and a decline in blood pH, with metabolic acidosis are other characteristic features of septic shock. ECG in the ICU can be used for hemodynamic monitoring. In patients with septic shock, cardiac output is usually increased with decreased peripheral resistance [15].

Data collection

Data were collected through retrospective review of patient medical records. Demographic details such as unique patient identifier number, age and sex of patient were extracted. GCS was monitored, and vital parameters, including heart rate, respiratory rate, and blood pressure were collected from available clinical documentation. Laboratory findings involving ABG measurements including lactate levels, CBC, CRP, renal function tests (serum creatinine), liver function tests (serum bilirubin) were mainly used for comprehensive assessment of sepsis. The SOFA score parameters involving PaO2/FiO2 or SpO2/FiO2 ratio, cardiovascular status determined by mean arterial pressure and vasopressor need, coagulation status were also taken into consideration for accurate diagnosis of sepsis and septic shock. The precise times of arrival to the ER and the administration of the first dose of antibiotics were recorded from medical records to assess treatment timelines.

Statistical analysis

Statistical analysis was performed with help of IBM SPSS Statistics for Windows, Version 20 (Released 2011; IBM Corp., Armonk, New York, United States). Continuous variables were tested for normality. As most variables were not normally distributed, data were presented as median (interquartile range or IQR) and comparisons between the sepsis without septic shock and septic shock groups were performed using the Mann-Whitney U test. Categorical variables are expressed as frequency (percentage) and were compared using Pearson’s chi-square test. Statistical significance was defined as a p-value of less than 0.05. All statistical tests conducted were two-tailed.

## Results

A total of 95 patients were included in the study, consisting of 56 male patients (58.9%) and 39 female patients (41.0%), as shown in Table 1.

**Table 1:** Gender of the study subjects

Gender	Frequency	Percentage
Male patients	56	58.9%
Female patients	39	41.0%

The association between sex and clinical group (septic shock and sepsis without septic shock) was assessed using Pearson’s chi-square test (Table 2).

**Table 2:** Association between sex and clinical group (septic shock and sepsis without septic shock) X^2 ^value: Pearson's chi-square; The association between two groups were performed using Pearson's chi-square statistical test.

Sex	Group			p-value
	Septic shock	Sepsis without septic shock	Χ^2 ^value	
Male patients (n=56)	12 (21.4%)	44 (78.6%)	6.467	0.011
Female patients (n=39)	18 (46.2%)	21 (53.8%)		
Total (n=95)	30 (31.6%)	65 (68.4%)		

The cohort consisted of 56 male subjects (58.9%) and 39 female subjects (41.0%), with an age range of 19 to 88 years and a mean age of 60.99 ± 15.14 years. The mean vital parameters recorded were temperature 99.23 °F ± 1.52, respiratory rate 28.76 ± 10.30 breaths/minute, systolic blood pressure 113.83 ± 26.30 mmHg, and GCS score 11.69 ± 3.34. the mean lactate levels was elevated 3.75 ± 2.74 mmol/L, indicating significant sepsis severity in the study population. The descriptive statistics of study population is given in Table 3 respectively.

**Table 3:** Statistical description of study population °F: Fahrenheit; bpm: breaths per minute; GCS: Glasgow coma scale.

Parameter	N	Minimum	Maximum	Mean ± SD
Age (years)	95	19	88	60.99 ± 15.14
Temperature (°F)	95	95.8	105	99.23 ± 1.52
Respiratory rate (bpm)	95	16	95	28.76 ± 10.30
GCS	95	2	15	11.69 ± 3.34.
Systolic BP (mmHg)	93	60	180	113.83 ± 26.30
Lactate (mmol/L)	94	0.6	23	3.75 ± 2.74
Time to antibiotic administration (min)	95	10	420	82.53 ± 70.58

Among these patients, 65 (68.4%) were diagnosed with sepsis without septic shock, and 30 (31.6%) progressed to septic shock during their admission in emergency department (Table 4).

**Table 4:** Patients with clinical diagnoses of septic shock and sepsis without septic shock

Type	Frequency	Percentage
Septic shock	30	31.6%
Sepsis without septic shock	65	68.4%

The median age, heart rate, respiratory rate, and time to antibiotic administration did not differ significantly between patients with septic shock and those with sepsis without septic shock (p>0.05 for all). In contrast, patients with septic shock had a significantly lower median GCS score and systolic blood pressure compared with patients without it (p=0.033 and p=0.017, respectively). Serum lactate levels were significantly higher among patients with septic shock than those without it (p<0.001), indicating greater severity of illness in the former group. A comparison of baseline clinical and laboratory parameters between patients with septic shock and sepsis without septic shock is shown in Table 5.

**Table 5:** Comparison of continuous variables between the septic shock and sepsis without septic shock groups Data are presented as median (IQR). IQR: interquartile range; Comparisons were performed using the Mann-Whitney U test (U-statistic value); br/min: breaths per minute; bpm: beats per minute; mmHg: millimeters of mercury; mmol/L: millimoles per liter; GCS: Glasgow coma scale.

Variable	Septic shock (n=30)	Sepsis without septic shock (n=65)	U-statistic value	p-value
	Median (Q_1_-Q_3_)	Median (Q_1_-Q_3_)		
Age (years)	64 (49.75-70)	62.5 (51-71.75)	965	0.936
Heart rate (bpm)	99 (87.5-119.75)	98(81.25-109.5)	779	0.463
Respiratory rate (br/min)	28 (24-32)	26(22-30.75)	942.5	0.794
GCS	12 (8-14.25)	13(12-14)	712	0.033
Systolic blood pressure (mmHg)	90 (80-130)	115(100-130)	627	0.017
Lactate level (mmol/L)	3.9 (3.1-5.15)	2.75(2-3.7)	455	<0.001
Time to antibiotic administration (min)	50 (39.5-120)	75 (40-135)	585	0.35

In patients who were diagnosed with septic shock, 20 patients (67%) received antibiotic therapy within 60 minutes, eight patients (26.5%) received therapy between 60 and 120 minutes and two patients (7.3%) were administered antibiotics after 120 minutes. The mean time to administration in this group was 61.52 minutes. Among patients diagnosed with sepsis without septic shock, 30 patients (46%) received antibiotics within 60 minutes of ER arrival. Fourteen patients (21.5%) were treated between 60 and 120 minutes, and 21 patients (32.3%) received antibiotics after 120 minutes. Here, the mean time to administration of antibiotics was 92.23 minutes (Table 6).

**Table 6:** Timing of antibiotic administration

Types	Time	Frequency	Percentage	Mean time (min)
Sepsis without septic shock	<60 minutes	30	46.0%	92.23
	60-120 minutes	14	21.5%	
	>120minutes	21	32.3%	
Septic shock	<60 minutes	20	67.0%	61.52
	60-120 minutes	8	26.5%	
	>120minutes	2	7.3%	

To evaluate the association between the timing of antibiotic administration and clinical outcomes was stratified across different antibiotic groups as show in Table 7.

**Table 7:** Association of antibiotic administration with mortality

Timing of antibiotic administration	Total patients (n)	Deaths (n)	Survivors (n)	Mortality (%)
<60 minutes	50	18	32	36.0%
60-120 minutes	22	11	44	50.0%
>120 minutes	23	18	5	78.30%
Total	95	47	48	49.5%

Mortality differed across antibiotic timing groups, with higher mortality observed in patients receiving antibiotics after 120 minutes compared to those treated within 60 minutes. A chi-square test showed a significant association between timing of antibiotic administration and mortality (χ²= 11.26, df=2, p=0.04). Mortality increased significantly with delay in antibiotic administration.

Subgroup analysis demonstrated an increasing trend in mortality with delayed administration in patients from septic shock and sepsis without septic shock groups. In patient with sepsis without septic shock, mortality increased from 26.7% in those receiving antibiotics within 60 minutes to 50% in the 60-120 minute group and 71.4% in those receiving antibiotics after 120 minutes. Similarly, in patients with septic shock, mortality increased from 50% (<60 minutes) to 62.5% (60-120 minutes) and reached 100% in patients receiving antibiotics after 120 minutes. This trend demonstrated a consistent increase in mortality with delayed antibiotic administration across both groups. The association between delayed antibiotic administration and mortality remained statistically significant in both groups (p<0.05), as shown in Table 8.

**Table 8:** Subgroup analysis of the timing of antibiotic administration and mortality in both groups

Group	Timing of antibiotic administration	Total (n)	Deaths (n)	Survivors (n)	Mortality (n)
Sepsis without septic shock	<60 minutes	30	8	22	26.7%
	60-120 minutes	14	7	7	50.0%
	>120 minutes	21	15	6	71.4%
Subtotal		65	30	35	46.2%
Septic shock	<60 minutes	20	10	10	50.0%
	60-120 minutes	8	5	3	62.5%
	>120 minutes	2	2	0	100%
Subtotal		30	17	13	56.7%
Total		95	47	48	49.5%

The most commonly used antibiotics for treatment of septic shock and sepsis without septic shock included meropenem (n=32, 33.7%), Piperacillin-tazobactam (n=27, 28.4%) and ceftriaxone (n=19, 20%).Other antibiotics included cefoperazone, clindamycin, azithromycin, doxycycline, and other combinations (Table 9).

**Table 9:** Frequency of antibiotic usage

Antibiotic	Frequency	Percentage
Ampicillin	1	1.1
Azithromycin	2	2.1
Ceftriaxone	19	20.0
Ciprofloxacin	1	1.1
Clindamycin	3	3.2
Doxycycline	2	2.1
Cefoperazone	6	6.3
Meropenem	32	33.7
Metrogyl	1	1.1
Piperacillin	27	28.4
Piperacillin + Clindamycin	1	1.1

The study also underscores the prognosis results of patients with septic shock and sepsis without septic shock, as assessed from in-hospital outcomes. Out of the total 95 patients, 47 (49.5%) died during hospitalization, while 48 (50.5%) were discharged after recovering completely.

In summary, patients with septic shock received antibiotics significantly earlier than those with sepsis without septic shock, reflecting adherence to treatment protocols in more critically ill patients. However, delays beyond 60 minutes were still observed in a considerable proportion of the overall cohort, specifically among those suffering from sepsis without septic shock. The overall mean time to antibiotic administration was 82.53 minutes. These findings underscores the ongoing need to improve timely antibiotic delivery in all sepsis-related cases to potentially reduce morbidity and mortality.

## Discussion

The findings from this study evaluated the relationship between the timing of antibiotic administration and clinical outcomes in patients with septic shock and those with sepsis without septic shock. Our findings demonstrate a statistically significant association between delayed antibiotic administration and increased in-hospital mortality. Mortality showed a clear increasing trend with delay, rising from 36.0% in patients receiving antibiotics within 60 minutes to 78.3% in those treated after 120 minutes. This establishes a time-dependent relationship between antibiotic administration and survival outcomes.

These findings are consistent with the current SSC 2021 guidelines, which recommend starting broad-spectrum antibiotics within one hour for septic shock and within three hours for sepsis without septic shock [10]. In our study, 67% (n=20) of patients with septic shock received antibiotics within 60 minutes; only 46% (n=30) of patients with sepsis without septic shock achieved this target. This disparity in antibiotic administration timing is a global challenge observed in many studies. Seymour et al. (2017) observed that hourly delays in the delivery of antibiotics were linked to higher in-hospital mortality, especially in patients suffering from shock [16]. Similarly, Liu et al. (2017) found that the death rate for patients with septic shock increased by 7.6% for every hour of delay in antibiotic therapy [9]. Our study reinforces these recommendations within an Indian emergency care setting, highlighting the clinical relevance of adhering to guideline-directed early antibiotic administration.

The subgroup analysis further strengthens this association. In both the septic shock and sepsis without septic shock groups, mortality increased progressively with the delay in antibiotic administration. Notably, in the septic shock subgroup, mortality reached 100% in patients receiving antibiotics after 120 minutes. This emphasizes that although patients with septic shock often receive earlier treatment, when delays occur, they can have profound consequences due to the severity of illness.

Also, serum lactate levels can be an early indicator in patients with sepsis without septic shock. The elevated mean serum lactate levels of 3.75 ± 2.74 mmol/L in our study indicate the severity of systemic hypoperfusion. Elevated lactate levels are often associated with septic shock and worse outcomes. The lactate levels, specifically above >2 mmol/L, are indicative of adverse outcomes in sepsis, and values over 4 mmol/L are a strong criterion of mortality. A study conducted by Nguyen et al. (2004) found that lactate-guided therapy in early phases helped showcase improved outcomes by administration of timely fluid resuscitation and vasopressor initiation [17]. Our findings are also suggestive of the same; lactate can be used as an early prognostic marker and should be assessed early in all patients suspected of sepsis.

Neurological assessment via the GCS revealed a mean score of 11.69 ± 3.34. Lower GCS scores have a correlation with sepsis-induced encephalopathy, which results in an increased risk of mortality. Shankar-Hari et al. (2007) reported that altered mental status, with lower GCS scores are predictive of higher mortality rate in patients with sepsis [18]. This helps signify the importance of neurological assessment in the early administration of antibiotics in suspected patients.

Regarding the vital signs, the mean respiratory rate of 28.76 breaths/min and systolic blood pressure of 113.83 mmHg suggest that many patients were in early septic shock. A respiratory rate of less than 22 breaths/min is a major component of the quick Sequential Organ Failure Assessment (qSOFA) score, which is used to identify patients who are at risk of poor outcomes outside an ICU setting [19]. In our study, antibiotic initiation was done appropriately for all septic shock cases, however elevated respiratory rate in sepsis cases should also be taken into consideration for prompt intervention, before it precipitates to shock.

Antibiotic usage in our study followed a pattern with meropenem (n=32, 33.7%), piperacillin-tazobactam (n=27, 28.4%), and ceftriaxone (n=19, 20%). This line of therapy aligns with the Infectious diseases Society of America (IDSA) and SCC guidelines for empirical treatment of individuals who maybe suspected with mixed or gram-negative infections [10,20]. Our study also reported a total in-hospital mortality rate of 49.5% (n=47), which constitutes patients from both groups. This rate of mortality, however, is comparable to the mortality rates seen among hospitals in India. For instance, according to the SEPSIS-India registry, a prospective study involving 19 ICUs across India, reported an overall mortality up to 50.8% in patients with septic shock, comparable to the critically-ill patients with septic shock in our study [21]. Another sepsis mortality rate study conducted by Chatterjee et al., again in an ICU setting, revealed a hospital mortality rate of 63.6% in patients with sepsis, who had significantly poor outcomes [22].

Our study, thus, highlights the importance of timely antibiotic administration in patients with septic shock and sepsis without septic shock. Although compliance was relatively higher in septic shock cases, delays in patients with sepsis without septic shock may have contributed to increased mortality. Addressing this gap requires strengthening of triage protocols, improving early recognition of sepsis, and streamlining diagnostic workflows to ensure prompt treatment. The use of standardized early warning scores such as qSOFA and National Early Warning Score (NEWS) [23] at triage, along with focused emergency room training in sepsis management, can facilitate earlier intervention. Implementation of these measures may significantly improve clinical outcomes and reduce sepsis-related mortality. 

Limitations

This study should be interpreted in light of certain limitations. Although conducted retrospectively, it was performed at a single tertiary care center with a relatively small sample size (n=95), which may limit the generalizability of the findings to other healthcare settings. The observational study precludes establishing a causal relationship between timing of antibiotic administration and clinical outcomes. In addition, potential confounding factors such as source of infection, comorbid conditions, and severity scoring beyond SOFA were not adjusted for multivariable analysis. Outcomes were limited to in-hospital events and longer term follow-up was not evaluated.

## Conclusions

This study demonstrates a significant association between delayed antibiotic administration and increased mortality in patients with septic shock and those with sepsis without septic shock. Mortality increased progressively with delay, with the highest rates observed in patients receiving antibiotics after 120 minutes. Subgroup analysis confirmed a similar trend in both groups, with a more pronounced impact in the septic shock cohort. These findings underscore the importance of early antibiotic initiation, particularly within the first hour of presentation, in improving survival outcomes. Strengthening timely recognition and prompt treatment protocols in emergency settings is essential for reducing sepsis-related mortality. Patients receiving antibiotics after 120 minutes had significantly higher mortality, with more than four-fold increased odds of death compared to those treated within an hour. Moreover, this effect was more pronounced in patients with septic shock. These findings highlight the critical importance of early antibiotic initiation, especially within the first hour of presentation, to improve survival outcomes. This study thus highlights the urgent need to improve awareness among ER staff about the importance of prompt treatment protocols to reduce sepsis-related mortality.
